# Staphylococcal Gangrenous Cholecystitis: A Case Report

**DOI:** 10.1155/crgm/3341650

**Published:** 2026-04-27

**Authors:** Anandu Mathews Anto, George Sarin Zacharia, Maria Jaquez Duran, Dmitry Lvovsky

**Affiliations:** ^1^ Department of Pulmonary Medicine, BronxCare Health System, New York City, New York, USA, bronxcare.org; ^2^ Department of Internal Medicine, BronxCare Health System, New York City, New York, USA, bronxcare.org

**Keywords:** cholecystitis, gall bladder, gangrenous, *Staphylococcus aureus*, Tokyo guidelines

## Abstract

Gangrenous cholecystitis is a severe and potentially fatal complication of acute cholecystitis, typically resulting from ischemic necrosis of the gallbladder wall secondary to sustained obstruction and inflammation. We present a rare case of methicillin‐sensitive *Staphylococcus aureus*–associated gangrenous cholecystitis with bacteremia in an elderly female without identifiable risk factors for staphylococcal infection. The patient was treated with antibiotics and emergent percutaneous cholecystostomy. This case highlights the need to consider infrequent pathogens, such as *S. aureus*, in patients with severe cholecystitis, particularly when clinical deterioration persists despite standard empirical therapy. Early diagnosis and prompt multidisciplinary management remain the key to a favorable outcome in this medically complex patient.

## 1. Introduction

Cholelithiasis affects approximately 10%–15% of the general population, with around 10%–15% of these individuals developing acute calculus cholecystitis [1, 2]. Calculus cholecystitis results from gallstone impaction in the neck of the gallbladder (GB) or cystic duct. The two‐hit model for acute cholecystitis explains the pathogenesis, with the first hit being GB outflow obstruction and the second GB hypoperfusion and inflammation [3]. Obstruction increases intraluminal pressure, resulting in venous congestion, impaired arterial and lymphatic flow, and subsequent inflammation. Prostaglandins are released, causing mucosal injury, while phospholipase activity converts lecithin into lysolecithin, a detergent‐like substance that disrupts the mucosa and produces ulcers with focal wall necrosis [4]. Gangrenous cholecystitis (GC) is a severe form of acute cholecystitis, qualifying as Tokyo Grade II severity, and is preferably diagnosed with computed tomography (CT) or magnetic resonance imaging (MRI) with contrast. It is associated with higher morbidity and mortality [5]. Gram‐negative Enterobacteriaceae are most frequent pathogens implicated in acute cholecystitis, most frequently in *Escherichia coli* and *Klebsiella pneumoniae* [6]. Gram‐positive bacteria are rarely isolated, most common being *Enterococcus* species [6]. *Staphylococcus*, though described, is rarely implicated in the patients with acute cholecystitis. Skorochod et al. reported a total of 28 patients with bile cultures positive for *Staphylococcus aureus*, over a period of 14 years; 17 of them were methicillin‐resistant [7]. The published literature reports *S. aureus* isolation rates ranging from 0.8% to 5.6% of patients with acute cholecystitis; confirming the microbiological rarity [7, 8].

## 2. Case Presentation

A 72‐year‐old female presented to the emergency department with severe abdominal pain and drowsiness. The patient’s family gave a history that she had been complaining of intermittent postprandial right upper quadrant abdominal pain for the past 2 months, which worsened over the past 1 week. She had reported nausea, vomiting, and worsening right upper quadrant pain. Her past medical history included congestive heart failure, hypertension, hypothyroidism, and gastroesophageal reflux disease. Her regular medications included aspirin, metoprolol, thyroxine, empagliflozin, losartan, and pantoprazole. On arrival, she was alert and oriented; Glasgow Coma Scale was 15, body temperature 100.3 F, pulse rate 123/min, respiratory rate 18/min, blood pressure 135/87 mm Hg, and oxygen saturation 96% on room air. She appeared in distress due to abdominal discomfort, with right upper quadrant tenderness on examination.

**FIGURE 1:**
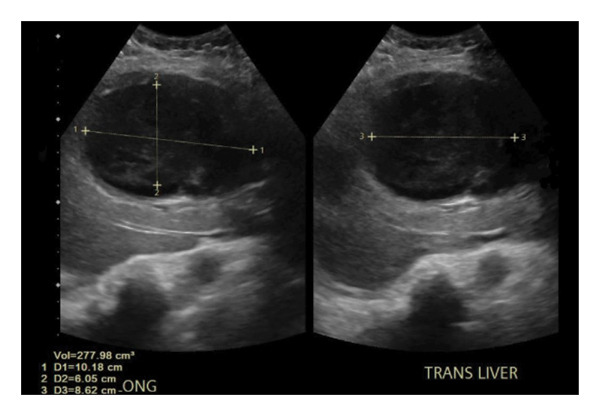
Ultrasound abdomen images demonstrating a markedly distended gallbladder with a thickened wall, heterogeneous internal echoes, and a pericholecystic collection.

**FIGURE 2:**
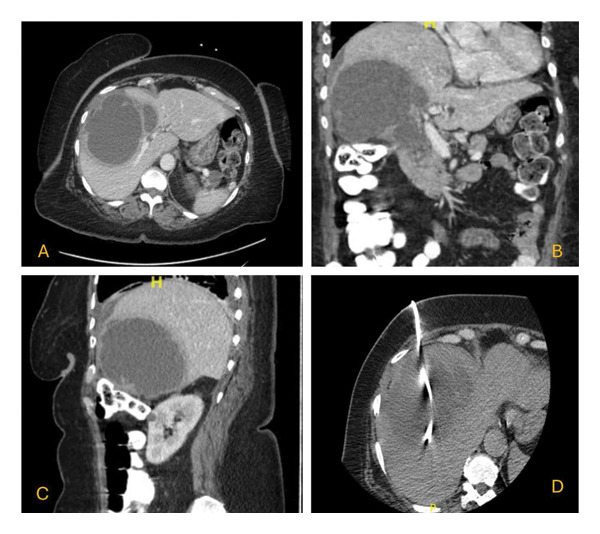
Contrast‐enhanced computed tomography images of the abdomen: (A) axial, (B) coronal, and (C) sagittal views revealing grossly distended gall bladder with wall thickening, pericholecystic fluid, and edema of the adjacent hepatic parenchyma. (D) Axial image following percutaneous image–guided cholecystostomy placement.

Laboratory studies revealed leukocytosis with left shift, normocytic anemia, thrombocytosis, hyponatremia, acute kidney injury with a creatinine of 2.5 mg/dL, and transaminitis. A respiratory viral infection panel was negative. Table 1 provides a snapshot of the relevant laboratory workup at presentation. Abdominal ultrasound demonstrated an echogenic liver and a markedly distended GB with a thickened wall, heterogeneous internal echoes, and a pericholecystic collection, concerning for GC and GB empyema (Figure 1). CT abdomen and pelvis showed a grossly distended, gangrenous gall bladder measuring 10.6 cm × 10 cm × 11.1 cm, with wall thickening, pericholecystic fluid, and edema of the adjacent hepatic parenchyma (Figure 2). Chest imaging revealed no pulmonary infiltrates.

**TABLE 1:** A summary of laboratory evaluation at presentation.

Laboratory test	Result	Normal limits
Total leukocyte count	21.9 k/ul	4.8–10.8 k/ul
Hemoglobin	9.7 g/dL	12–16 g/dL
Platelet count	54 k/ul	150–400 k/ul
Sodium	133 mEq/L	135‐145 mEq/L
Potassium	4 mEq/L	3.5–5 mEq/L
Blood urea nitrogen	15 mg/dL	6–20 mg/dL
Creatinine	2 mg/dL	0.5–1.5 mg/dL
Bicarbonate	20 mEq/L	24‐30 mEq/L
Lactate	2.8 mmol/L	0.5–2 mmol/L
pH	7.32	7.35–7.45
Anion gap	17 mmoles/L	9–15 mmoles/L
Albumin	2.8 g/dL	3.2–4.6 g/dL
Total bilirubin	0.6 g/dL	0.2–1.1 mg/dL
Aspartate aminotransferase	47 unit/L	9–36 unit/L
Alanine aminotransferase	43 unit/L	5–40 unit/L
Total protein	6.8 g/dL	5.8–8.3 g/dL
HbA1c	5.7%	4.7%–6.4%
C‐reactive protein	56 mg/L	1.0–3.0 mg/L
Prothrombin time	12.6	11–13 s
aPTT	28	20–34 s

She was initiated on empirical antibiotic therapy: piperacillin–tazobactam, and underwent emergent interventional radiology–guided cholecystostomy tube placement, followed by admission to the intensive care unit. Preliminary blood cultures grew *S. aureus*, while the cholecystostomy samples revealed Gram‐positive cocci in clusters, prompting the addition of vancomycin. By Day 3, blood cultures confirmed methicillin‐sensitive *S. aureus* (MSSA) bacteremia. The intraprocedural cultures from the GB also grew MSSA. Vancomycin and piperacillin–tazobactam were discontinued, and she was transitioned to cefazolin plus ertapenem, the latter added for anaerobic and Gram‐negative coverage with reported synergy in MSSA bacteremia. The patient reported subjective improvement by the fifth day of hospitalization; however, leukocytosis persisted. On Day 7, she developed nonoliguric acute kidney injury with metabolic acidosis, likely secondary to sepsis‐related acute tubular necrosis. She was managed with intravenous fluids, oral sodium bicarbonate, and close monitoring of fluid and electrolyte status, in concurrence with the nephrology team; no renal replacement therapy was required. Transthoracic and transesophageal echocardiography were negative for infective endocarditis. The patient continued to improve clinically, and leukocyte counts started to drop serially from Day 10. A repeat CT of the abdomen and pelvis on Day 14 confirmed proper cholecystostomy tube position. Ertapenem was discontinued, and she was continued on intravenous cefazolin monotherapy. She became apparently asymptomatic, worked with physical therapy, and was discharged home on Day 19; she continued on intravenous cefazolin via home infusion services for a total duration of 4 weeks. At the surgical clinic follow‐up, she was completely asymptomatic, with the cholecystostomy tube draining 200–300 mL of bile daily. The cholecystostomy tube was removed subsequently; however, she refused an elective cholecystectomy. The patient was completely asymptomatic at 6‐month follow‐up.

## 3. Discussion

Acute cholecystitis is one of the leading causes of acute abdomen and is the most common complication of cholelithiasis. GC, a severe variant of acute cholecystitis, is characterized by gall bladder ischemia and necrosis, culminating in perforation. The incidence of GC ranges from 2% to 30% of acute cholecystitis cases [9]. The pathophysiology of GC involves increased GB intraluminal pressure, leading to increased GB wall strain, which in turn results in hypoperfusion and ischemic necrosis [9]. The suggested predictors of GC, although they vary between studies, include age over 50 years, male gender, diabetes mellitus, history of cardiovascular disease, delayed presentation, leukocytosis exceeding 14,000/mm^3^, and tachycardia exceeding 90 beats/minute [9–11]. GC is associated with a higher incidence of perforation, prolonged hospitalization, and higher mortality rates compared to uncomplicated cholecystitis [11].

The diagnosis of cholecystitis relies on a combination of clinical features and diagnostic imaging, as defined by the Tokyo Guidelines, 2018. A definitive diagnosis of acute cholecystitis requires the presence of both local and systemic inflammation, along with suggestive imaging features. The recommended features of local inflammation include right upper quadrant pain or tenderness (Murphy’s sign), while those of systemic inflammation include fever, elevated C‐reactive protein, or leukocytosis. Ultrasound is the recommended first‐line imaging modality for acute cholecystitis. The findings include sonographic Murphy’s sign, GB wall thickness ≥ 4 mm, an enlarged GB ≥ 8 cm in the long axis or ≥ 4 cm in the short axis, pericholecystic fluid, or fat stranding [5]. Ultrasound has a sensitivity of 81% and a specificity of 83% for diagnosing acute cholecystitis [12]. However, in cases of GC, Murphy’s sign may be absent due to possible denervation of the GB wall [13]. Additional imaging modalities for diagnosis include CT, MRI, or a hepatobiliary iminodiacetic acid (HIDA) scan, which is the gold standard. Contrast CT or MRI is recommended over ultrasound for the diagnosis of GC; the findings include gas in the wall or lumen, intraluminal membranes, irregular wall thickening, pericholecystic abscess, and poor contrast enhancement of the GB wall [5]. The greater the degree of GB distension in the short axis and wall thickening, the greater the likelihood of GC [14].

The Tokyo consensus grades acute cholecystitis based on the severity: Grade III with organ/system dysfunction; Grade II with local complications, leukocytosis > 18,000//mm3, or total duration more than 72 h; Grade I: cholecystitis not meeting the criteria for higher grades [5]. In patients with Grade II and III acute cholecystitis, antibiotics and general supportive measures should be initiated promptly. Patients with a favorable risk profile could be candidates for early laparoscopic cholecystectomy, provided advanced techniques and expertise are available. Alternatively, they could be initially managed with urgent/early gall bladder drainage, followed by elective cholecystectomy [5].

Our patient fulfilled the Tokyo guidelines criteria for Grade III acute cholecystitis as she had GC with altered mental status and acute kidney injury with a creatinine of more than 2 mg/dL. Contrast abdominal CT revealed GC with a distended GB, high‐attenuation luminal bile, an intraluminal membrane, and pericholecystic changes. Her initial APACHE score was 15, the Charlson comorbidity index was 5, the American Society of Anesthesiologists physical status was 3, and the total bilirubin level was 0.6 mg/dL, suggesting poor surgical candidacy, especially in a secondary‐level hospital setting [15]. She was initiated on Gram‐negative and anaerobic bacterial coverage and underwent percutaneous cholecystostomy for biliary drainage. Interestingly, her blood culture and bile culture grew *S. aureus*. According to the literature, the most common organisms isolated in acute cholecystitis are *E. coli*, *Klebsiella*, *Enterococcus*, *Enterobacter*, and anaerobes such as *Clostridium* and *Bacteroides* [16]. Staphylococcal biliary infections are exceedingly uncommon.

A database analysis conducted at a high‐volume tertiary care hospital in Israel identified only 28 cases of Staphylococcal biliary infections over 14 years [7]. Other studies have also reported a low incidence of *S. aureus* isolation from bile, with rates ≤ 2% [17, 18]. A concise summary of reports/literature on *S. aureus* cholecystitis is tabulated as Table 2. Our patient denied intravenous drug abuse, recent hospitalizations, and surgical interventions, and evaluation for alternate source(s) of Staphylococcal bacteremia, including echocardiography, was noncontributory. However, she was a resident of the Bronx borough of New York, where the incidence of community‐acquired Staphylococcal infections, including methicillin‐resistant variants, is relatively high [25]. Intravenous vancomycin was initiated for Staphylococcal bacteremia and later switched to cefazolin after the organism was determined to be methicillin‐susceptible. The patient responded to management, with improvement in clinical and laboratory parameters, and was subsequently discharged.

**TABLE 2:** A summary of published literature on acute *Staphylococcus aureus* cholecystitis.

Publication geographic location	Patient profile			Methicillin susceptibility	Complications	Outcome
	Age (years)	Gender	Risk factors and comorbidities			
Skorochod et al. (28 patients) [7] Jeruselem, Israel	62.2 ± 19	M 15; F 13	DM (25%); CKD (25%); Hypertension (57.1%); BPH (25%); Liver disease (7.1%); Immunosupression (10.7%); Bed‐ridden (14.3%)	MRSA: 60.7% MSSA: 39.3%	N/A	Discharge: 82.1% Death: 17.9%

Merchant et al. (3 patients) [18] Rochester, New York, USA	A. 64	F	Hypertension, hypothyroidism, cholelithiasis	MSSA	Delirum	Discharge
	B. 73	F	DM, hypertension, coronary artery diseases, atrial fibrillation, psoriasis, recent diverticular bleed and hip fracture, choleithiasis	MRSA	Renal failure, respiratory failure	Death
	C. 48	M	Cirrhosis, Hepatitis C, DM, Hypertension, Cholelithiasis	MRSA	Nil	Discharge

Kowsika et al. [19] Mckeesport, Pennsylvania, USA	73	F	Cirrhosis	N/A	Nil	Discharge

Choudhury et al. [20] Dhaka, Bangladesh	59	M	Pulmonary embolism (Past)	MRSA	Nil	Discharge

Hadano et al. [21] Tokyo, Japan	58	M	DM (new; diagnosed during hospitalization)	MSSA	Acute renal failure upper GI bleed cardiac arrest	Death

Nepal et al. [22] South Dakota, USA	50 s	F	DM, ethanol use, presenile dementia and seizures secondary to traumatic brain injury, cholelithiasis	MRSA	Seizures Altered mental status	Discharge

Kim et al. [23] Alberta, Canada	36	F	HIV, Hepatitis C, cholelithiasis	MRSA	Acute renal failure	Discharge

Yu et al. [24] California, USA	65	M	ESRD on hemodialysis, coronary artery disease, peripheral artery disease	MRSA	Hypotension Altered mental status	Death

Our patient Bronx, New York, USA	72	F	Hypertension, hypothyroidism, heart failure	MSSA	Acute renal failure	Discharge

*Note:* N/A: information not available.

Abbreviations: BPH, benign prostatic hypertrophy; CKD, chronic kidney disease; DM, diabetes mellitus; ESRD, end stage renal disease; F, female; M, male; MRSA, methicillin‐resistant *Staphylococcus aureus*; MSSA, methicillin‐sensitive *Staphylococcus aureus*.

## 4. Conclusion

GC is a severe form of acute cholecystitis linked with higher morbidity and mortality. We describe a rare case of community‐acquired, MSSA Grade III GC complicated by bacteremia, with no other apparent source of infection, which was managed successfully with early cholecystostomy and intravenous antibiotics. This case underscores the importance of rapid diagnosis, early source control, and pathogen‐directed antibiotic therapy in biliary tract infections.

## Funding

No funding was received for this manuscript.

## Conflicts of Interest

The authors declare no conflicts of interest.

## Data Availability

The data that support the findings of this study are available on request from the corresponding author. The data are not publicly available due to privacy or ethical restrictions.
